# Associations of physical activity and sedentary behavior with insomnia in middle-aged and older adults: a cross-sectional study

**DOI:** 10.3389/fpubh.2025.1700775

**Published:** 2025-12-17

**Authors:** Yiying Lu, Min Liu, Sijing Chen, Xiujun Liu

**Affiliations:** 1 Wuhan Children’s Hospital (Wuhan Maternal and Child Healthcare Hospital), Tongji Medical College, Huazhong University of Science and Technology, Wuhan, China; 2 Wuhan Mental Health Center, Wuhan, China

**Keywords:** a cross-sectional study, insomnia, middle-aged and older adults, physical activity, sedentary behavior

## Abstract

**Objective:**

To explore the relationships between physical activity (PA) and sedentary behavior (SB) levels and insomnia among middle-aged and older adults.

**Methods:**

This cross-sectional study involved 3,752 middle-aged and older adults aged 50 years or older from Wuhan, China. PA and SB levels were assessed via the International Physical Activity Questionnaire (IPAQ)-Short Form. Insomnia severity was evaluated by the Insomnia Severity Index (ISI). Multivariate logistic regression was employed to estimate the impact of PA and SB on insomnia.

**Results:**

A total of 3,752 middle-aged and older adults in Wuhan were included. Among them, 18.0% reported insomnia symptoms, with 13.6% classified as mild insomnia and 4.4% as moderate-to-severe insomnia. After adjustment for confounders, high levels of physical activity were found to be associated with lower odds ratios for mild insomnia than low levels of PA (OR (95% CIs) = 0.67 (0.50, 0.90)), whereas 3–6 h/day and ≥6 h/day of sedentary behavior were associated with higher odds ratios for mild insomnia than were <3 h/day of sedentary behavior (OR (95% CIs) = 1.41 (1.09, 1.83) and 1.71 (1.19, 2.45), respectively). Similarly, moderate and high levels of physical activity were associated with lower odds ratios for moderate insomnia (OR (95% CIs) = 0.45 (0.28, 0.72) and 0.47 (0.30, 0.73), respectively), whereas ≥6 h per day of sedentary behavior was associated with higher odds ratios for moderate insomnia (OR (95% CIs) = 2.47 (1.37, 4.45)).

**Conclusion:**

High PA levels were associated with a reduced risk of both mild and moderate-to-severe insomnia in middle-aged and older adults, while SB ≥ 3 h/day increased insomnia risk—with the highest risk observed at SB ≥ 6 h/day. Clinically, these findings support non-pharmacological interventions for insomnia prevention and management in this population: recommending achievement of high PA levels (e.g., ≥1,500 MET-minutes/week of vigorous activity or ≥3,000 MET-minutes/week of combined activity) and limiting daily SB to <3 h. Targeted PA/SB intervention programs should be prioritized for high-risk subgroups, such as women and low-income individuals.

## 1 Introduction

Insomnia is a prevalent sleep disorder characterized by persistent difficulties in falling asleep, maintaining sleep, or experiencing early morning awakening without the ability to resume sleep (1). These sleep disturbances can persist for months or even years, resulting in daytime impairments such as deficits in attention, concentration, and memory (2, 3). The estimated prevalence of insomnia in the general Chinese population is approximately 15% (4). Insomnia is frequently comorbid with psychiatric conditions, particularly depression (5, 6). In addition to mental health concerns, insomnia has been associated with an increased risk of cardiovascular diseases (7–9). Moreover, the role of insomnia in increasing suicide risk, both directly and indirectly through its association with mental disorders, has been well documented (10).

Given the serious health consequences of insomnia, the identification of modifiable risk factors is crucial. China faces a rapidly aging population, the prevalence of insomnia in Chinese middle-aged and older adults is significantly higher than in younger populations. Nationally, the prevalence of insomnia among adults ≥60 years ranges from 23.5 to 38.7%, with higher rates in rural areas (35.2%) than in urban areas (28.1%); insomnia severity also increases with age and decreases with educational attainment (11). Additionally, insomnia prevalence is higher in northern China (32.8%) than in southern China (25.6%), potentially due to climate-related increases in indoor sedentary time (12). Insomnia not only impairs quality of life in middle-aged and older adults but also directly increases the risk of cognitive decline and falls, imposing an annual medical burden of over 5 billion RMB in China—highlighting the public health significance of this study (13, 14). This group in urban China also experiences unique lifestyle challenges, such as high rates of sedentary behavior due to retirement or desk-based work, making them a critical population for studying PA-SB-insomnia relationships. Insomnia significantly impacts cognitive function and mental health in middle-aged and older adults. Increasing evidence suggests an association between physical activity (PA; defined as any bodily movement resulting in energy expenditure, including light, moderate, and vigorous intensity activities) and sedentary behavior (SB; defined as waking behavior characterized by low energy expenditure while sitting, reclining, or lying down, such as watching TV, desk work, or driving) with insomnia (15, 16). Prolonged SB is associated with insulin resistance and elevated inflammatory responses, which may exacerbate sleep disturbances and other chronic conditions (17). Previous studies have shown that PA exerts a protective effect on sleep by regulating the autonomic nervous system and improving emotional states, while SB may disrupt the sleep–wake cycle by reducing nighttime melatonin secretion (18). Ross R reported that physical activity is related to improved sleep quality among middle-aged and older adults (19). However, some studies, such as one conducted in Singapore, did not demonstrate a significant independent relationship between PA, SB, and sleep quality. Notably, the relationships among these factors remain uncertain (20).

Insomnia is highly comorbid with depression and anxiety: approximately 45.2% of middle-aged and older adults with insomnia have comorbid depressive symptoms, and 38.7% have comorbid anxiety symptoms, with the severity of insomnia positively correlated with depression/anxiety levels (21). Different opinions exist regarding the benefits of light versus vigorous physical activity. Some studies suggest that both types are beneficial; for example, one study reported that physical activity positively influences sleep quality (22). Conversely, other research indicates that vigorous physical activity may lack benefits, with a study showing that excessive activity can lead to fatigue, adversely affecting sleep (23). The current literature explores the relationships between PA, SB, and insomnia; however, the dose–response relationships among these factors have not been addressed. Therefore, further investigations are needed to examine the potential dose–response relationships between PA, SB, and insomnia in a large, representative sample of middle-aged and older adults experiencing sleep disturbances and reduced activity levels.

The primary objective of this study was to evaluate the dose–response relationships between physical activity, sedentary behavior, and insomnia in middle-aged and older Chinese adults. By examining how varying levels of PA and SB influence the severity of insomnia, this research aims to provide clearer insights into the modifiable factors that could inform effective interventions for preventing and managing insomnia in aging populations.

## 2 Methods

A cross-sectional study was conducted in 2021 to collect data from middle-aged and older Chinese adults through a household survey. A stratified random sampling method was used. Wuhan’s administrative divisions were stratified into urban districts (e.g., Wuchang, Jianghan) and suburban districts (e.g., Jiangxia, Huangpi) on the basis of the Wuhan Municipal Bureau of Statistics registry. Within each stratum, communities were randomly selected in proportion to their population size, and eligible participants (aged 50 years or older, with normal cognition) were randomly selected from household registration lists and invited to participate via telephone and home visits. The inclusion criteria were as follows: ① Chinese middle-aged and older individuals aged 50 years and older; ② Screening for normal cognitive ability was conducted using the Mini-Mental State Examination (MMSE). Participants should possess normal cognitive abilities. ③ Voluntary signing of the informed consent form. One-on-one questionnaire surveys were administered to eligible participants by trained researchers. A total of 3,752 middle-aged and older adults were included in the study.

### 2.1 Dependent and independent variables

In this study, insomnia was the dependent variable and was assessed via the Insomnia Severity Index (ISI). The ISI evaluates subjective symptoms, daily functioning interference, quality of life, and distress caused by sleep problems over the past month (24, 25). The Insomnia Severity Index has been validated for use in Chinese populations, demonstrating good internal consistency (Cronbach’s *α* = 0.87) and convergent validity with clinical diagnoses of insomnia (26). The ISI is a self-report questionnaire consisting of seven items, each scored on a 5-point Likert scale ranging from 0 to 4. The total score ranges from 0 to 28, with higher scores indicating more severe insomnia. The outcome variables of insomnia were categorized as follows: no clinically significant insomnia (0–7 points), subthreshold insomnia (8–14 points), moderate insomnia (15–21 points), and severe insomnia (22–28 points). For this study, moderate (15–21) and severe (22–28) insomnia were combined into a “moderate to severe insomnia” group because of their clinical similarity in intervention protocols (27).

PA and SB are defined based on the assessment criteria of the International Physical Activity Questionnaire (IPAQ) short form (28). The International Physical Activity Questionnaire-Short Form has also been validated in Asian adults and has moderate to strong reliability (test–retest ICC = 0.62–0.81) and validity in estimating physical activity levels (29). PA refers to any bodily movement that results in energy expenditure, encompassing activities ranging from light daily tasks to vigorous exercise. The frequency and duration of walking, moderate-intensity activities, and vigorous activities over the past week were collected from participants via the IPAQ. The metabolic equivalent (MET) ranges for different activity types were established, and the median was calculated. The activity frequency was multiplied by the duration (converted to hours) and then by the median MET value to obtain MET-minutes/week for each activity type, enabling the calculation of overall physical activity (PA). High PA levels were defined on the basis of the International Physical Activity Questionnaire (IPAQ) criteria as follows: vigorous activity: at least 1,500 MET-minutes/week from vigorous-intensity activities (e.g., running, swimming) performed on ≥3 days/week. Combined activities: At least 3,000 MET-minutes/week from a combination of vigorous-intensity activities, moderate-intensity activities (e.g., brisk walking, cycling), and/or walking, accumulated over ≥7 days/week. Moderate PA levels were defined as vigorous activity for at least 20 min/day of vigorous-intensity activity >3 days/week (total ≥60 min/week). Moderate-intensity activity was defined as at least 30 min/day of moderate-intensity activity for ≥5 days/week (total ≥150 min/week). Combined walking and other activities: At least 30 min/day of walking combined with moderate/vigorous activities on ≥5 days/week, totaling ≥600 MET-minutes/week.

Individuals not meeting the criteria for high or moderate PA were classified as having low PA levels (30). SB was assessed as average daily sedentary time (including weekdays and weekends), with cutoffs of <3, 3–6, and ≥6 h/day, on the basis of prior studies in middle-aged and older adults defining prolonged sedentary behavior as ≥3 h/day.

### 2.2 Sociodemographic, biological, behavioral and psychological factors

Sociodemographic characteristics such as sex, age, BMI, education level (categorized as primary school and below, middle school, and high school and above), marital status, and monthly family income (categorized as less than 3,000 RMB, 3000–−5999 RMB, 6000–−10000 RMB, or 10,000 RMB and above) were recorded. Physical health-related factors, including self-reported chronic pain persisting or recurring for more than 3 months, as well as the number of chronic diseases (0, 1, or ≥2), were captured. Behavioral factors were assessed on the basis of self-perceived quality of life, which was measured via the QOL-6, and whether participants were current drinkers or smokers. Depressive symptoms were assessed using the Patient Health Questionnaire-9 (PHQ-9), which demonstrates excellent reliability and validity in Chinese populations (Cronbach’s *α* = 0.85, test–retest reliability ICC = 0.82) and effectively identifies depressive states in middle-aged and older adults (31). Anxiety symptoms were evaluated using the Generalized Anxiety Disorder-7 (GAD-7), with Cronbach’s *α* = 0.83 and a Kappa coefficient of 0.76 for agreement with clinical diagnoses—confirming its suitability for this population (32).

### 2.3 Statistical analysis

In this study, descriptive analyses of the demographic variables were conducted, with <5% missing data for all the variables. Incomplete questionnaires were excluded, and no imputation was performed. The data are presented as the means, standard deviations, and percentages. Chi-square tests, Kruskal–Wallis H tests, or one-way ANOVA were used to compare the characteristics of multiple groups of subjects. Continuous variables with a normal distribution are presented as the means ± SDs and were compared via one-way ANOVA; nonnormally distributed continuous variables are presented as medians (IQRs) and were compared via the Kruskal–Wallis H test. Categorical variables are presented as counts (%) and were compared via the chi-square test. Multivariate logistic regression analysis was employed to examine the relationships among PA levels, SB levels, and insomnia.

Three models were used in the analysis. In Model 1, PA levels and SB levels were considered independent variables for estimating unadjusted associations. In Model 2, adjustments were made for potential confounders such as sex, age, BMI, education level, marital status, monthly family income, smoking status, and drinking status. In Model 3, further adjustments were incorporated for the presence of chronic diseases (no/yes). Odds ratios (ORs) with 95% confidence intervals (CIs) are reported.

To further examine the results in subgroups, stratified analyses were performed on the basis of sex, education level, monthly family income, smoking status, and drinking status. Stratified analyses were performed for two outcomes: mild insomnia (8–14 points) and moderate–to-severe insomnia (15–28 points), each compared with no insomnia (0–7 points). To examine the potential moderating role of variables on the associations between PA/SB and insomnia, interaction effect analysis was conducted based on Model 3: (1) Continuous variables (e.g., PA, SB) were centered; (2) Interaction terms were constructed, including gender×PA, gender×SB, educational level×PA, educational level×SB, monthly income×PA, monthly income×SB, smoking×PA, smoking×SB, drinking×PA, and drinking×SB; (3) Each interaction term was incorporated into Model 3, and multivariate logistic regression was used to calculate the regression coefficient (B), Wald statistic, odds ratio (OR), and 95% confidence interval (CI) of interaction effects. The data were analyzed via IBM SPSS Statistics 26.0. A *p* value < 0.05 was considered statistically significant.

### 2.4 Ethical considerations

This study is a secondary data analysis, with raw data derived from a previously published study (33). The original study was approved by the Ethics Committee of Wuhan Mental Health Center, and the protocol for secondary analysis was reported to and approved by the same committee. The participants provided written informed consent to participate in this study.

## 3 Results

### 3.1 Sample characteristics

The sociodemographic, biological, behavioral, and psychological characteristics of the study sample are summarized in Table 1. Among the participants, 18% were found to experience insomnia, with 13.6% classified as having mild insomnia and 4.4% as having moderate to severe insomnia. Compared with the non-insomnia group, participants with mild and moderate to severe insomnia demonstrated significantly lower levels of physical activity. The median weekly physical activity levels were 3,066 (IQR: 1386–5040) MET-minutes/week in the non-insomnia group, 2,373 (IQR: 1168–4746) MET-minutes/week in the mild insomnia group, and 1737 (IQR: 693–4746) MET-minutes/week in the moderate-to-severe insomnia group (*p* < 0.001). Similarly, sedentary behavior was notably greater among participants with insomnia symptoms. The average daily sedentary time was 3.36 ± 1.84 h (mean±standard deviation) for the non-insomnia group, increasing to 3.55 ± 1.99 h for the mild insomnia group and 3.90 ± 2.59 h for the moderate to severe insomnia group (*p* < 0.001).

**Table 1:** Participants’ characteristics and comparisons among different groups of insomnia.

Characteristics	No insomnia (*n* = 3,075)	Mild insomnia (*n* = 511)	Moderate to severe insomnia (*n* = 166)	*p*
Male, *n* (%)	1,128 (36.68)	113 (22.11)	33 (19.88)	<0.001
Age (y)	63.95 (57.71–70.09)	65.15 (58.37–71.41)	65.50 (60.19–71.90)	0.002
BMI (kg/m^2^)	23.52 ± 3.23	23.25 ± 3.28	22.68 ± 3.55	0.002
Education, *n* (%)				<0.001
Primary school and below	1,196 (38.89)	257 (50.29)	103 (62.05)	
Middle school	996 (32.39)	139 (27.20)	39 (23.49)	
High school and above	883 (28.72)	115 (22.50)	24 (14.46)	
Marital status, *n* (%)				<0.001
Married	2,668 (86.76)	404 (79.06)	131 (78.91)	
Others	407 (13.24)	107 (20.94)	35 (21.08)	
Monthly family income, *n* (%)				<0.001
<3,000 RMB	903 (29.37)	203 (39.73)	78 (46.99)	
3,000–5,999 RMB	719 (23.38)	113 (22.11)	37 (22.29)	
6,000–10,000 RMB	887 (28.85)	120 (23.48)	29 (17.47)	
≥10,000 RMB	66 (18.41)	75 (14.68)	22 (13.25)	
Current smoker, *n* (%)	518 (16.85)	68 (13.31)	18 (10.84)	0.022
Current drinker, *n* (%)	430 (13.98)	42 (8.22)	5 (3.01)	<0.001
Chronic pain, *n* (%)	737 (23.97)	288 (56.36)	105 (63.25)	<0.001
Chronic disease, *n* (%)				<0.001
0	1,504 (48.91)	158 (30.92)	37 (22.29)	
1	1,042 (33.89)	200 (39.14)	60 (36.14)	
≥2	529 (17.20)	153 (29.94)	69 (41.57)	
Physical activity (MET-min/w)	3,066 (1,386–5,040)	2,373 (1,168–4,746)	1737 (693–4,746)	<0.001
Physical activity levels, *n* (%)				<0.001
Low	371 (12.07)	85 (16.63)	41 (24.70)	
Medium	1,106 (35.97)	197 (38.55)	51 (30.72)	
High	1,598 (51.97)	229 (44.81)	74 (44.58)	
Sedentary time (hour/d)	3.36 ± 1.84	3.55 ± 1.99	3.90 ± 2.59	<0.001
Sedentary behavior, *n* (%)				<0.001
<3 h	764 (24.85)	95 (18.59)	36 (21.69)	
3–6 h	1979 (64.36)	341 (66.73)	96 (57.83)	
≥6 h	332 (10.80)	75 (14.68)	34 (20.48)	
PHQ-9 score	0.88 ± 1.84	4.22 ± 3.66	7.75 ± 5.76	<0.001
GAD-7 score	0.50 ± 1.43	2.30 ± 3.24	4.43 ± 5.14	<0.001
QOL-6 score	24.99 ± 3.48	21.76 ± 3.35	19.55 ± 3.55	<0.001
ISI score	1.58 ± 2.10	10.55 ± 2.04	17.65 ± 2.82	<0.001

Continuous variables with a normal distribution (age, BMI) were compared via one-way ANOVA; non-normal variables (PA, sedentary time) were compared via the Kruskal–Wallis H test; categorical variables were compared via the chi–square test.

In terms of sociodemographic characteristics, participants with moderate to severe insomnia were more likely to have lower educational levels, with 62.05% having primary education or below, than were 38.89% in the non-insomnia group (*p* < 0.001). A similar trend was observed for monthly family income, where 46.99% of participants with moderate to severe insomnia reported earning less than 3,000 RMB per month, compared with 29.37% in the non-insomnia group (*p* < 0.001). Additionally, individuals with moderate to severe insomnia had a greater prevalence of chronic pain (63.25%) and multiple chronic diseases (41.57%) than did those without insomnia symptoms (23.97 and 17.20%, respectively; *p* < 0.001).

### 3.2 Regression analysis

Table 2 presents the associations between physical activity, sedentary behavior, and insomnia, with consistent patterns observed across all three models (34). In Model 1 (unadjusted), higher levels of physical activity were significantly linked to a reduced risk of both mild (OR = 0.68, 95% CI: 0.51–−0.90) and moderate-to-severe insomnia (OR = 0.47, 95% CI: 0.31–−0.70). In contrast, sedentary behavior, particularly sitting for more than 6 hours per day, was associated with an increased risk of mild (OR = 1.65, 95% CI: 1.18–2.31) and moderate to severe insomnia (OR = 1.87, 95% CI: 1.13–3.09).

**Table 2:** Associations between physical activity, sedentary behavior and insomnia.

	Physical activity				Sedentary behavior			
	Low	Moderate	High	*p*	<3 h	3–6 h	≥6 h	*p*
Model 1								
Mild insomnia	1	0.80 (0.61, 1.06)	**0.68 (0.51, 0.90)**		1	**1.33 (1.04, 1.70)**	**1.65 (1.18, 2.31)**	
Moderate to severe insomnia	1	**0.44 (0.29, 0.67)**	**0.47 (0.31, 0.70)**	<0.001	1	0.97 (0.65, 1.44)	**1.87 (1.13, 3.09)**	<0.001
Model 2								
Mild insomnia	1	0.85 (0.64, 1.13)	**0.67 (0.51, 0.89)**		1	**1.37 (1.07, 1.75)**	**1.69 (1.20, 2.39)**	
Moderate to severe insomnia	1	**0.50 (0.32, 0.77)**	**0.47 (0.31, 0.72)**	<0.001	1	1.03 (0.69, 1.54)	**2.04 (1.21, 3.43)**	<0.001
Model 3								
Mild insomnia	1	0.84 (0.62, 1.13)	**0.67 (0.50, 0.90)**		1	**1.41 (1.09, 1.83)**	**1.71 (1.19, 2.45)**	
Moderate to severe insomnia	1	**0.45 (0.28, 0.72)**	**0.47 (0.30, 0.73)**	<0.001	1	1.46 (0.92, 2.33)	**2.47 (1.37, 4.45)**	<0.001

Model 1 was unadjusted. Model 2 was adjusted for sex, age, BMI, education, marital status, monthly family income, smoking status, and drinking status. Model 3 was further adjusted for chronic disease (yes or no). The bold values indicate statistical significance (*p* < 0.05).

After adjusting for sex, age, BMI, education, marital status, monthly income, smoking, and drinking habits in Model 2, high physical activity continued to have a protective effect against both mild (OR = 0.67, 95% CI: 0.51–−0.89) and moderate-to-severe insomnia (OR = 0.47, 95% CI: 0.31–−0.72). Participants with sedentary behavior of≥6 h per day had a higher risk of mild insomnia (OR = 1.69, 95% CI: 1.20–2.39) and moderate to severe insomnia (OR = 2.04, 95% CI: 1.21–3.43) than those with sedentary behavior of ≤6 h.

In Model 3, which included further adjustments for chronic disease status, similar trends were observed. High levels of physical activity were consistently correlated with a lower risk of mild (OR = 0.67, 95% CI: 0.50–−0.90, *P*<0.05) and moderate-to-severe insomnia (OR = 0.47, 95% CI: 0.30–−0.73, *p* < 0.001). Moreover, prolonged sitting continued to be a significant predictor of insomnia, with the risk of mild (OR = 1.71, 95% CI: 1.19–−2.45, *p* < 0.01) and moderate-to-severe insomnia (OR = 2.47, 95% CI: 1.37–−4.45, *p* < 0.01) being elevated.

Table 3 shows a stratified analysis of the effects of physical activity and sedentary behavior on mild insomnia (8–14 points) and moderate-to-severe insomnia (15–28 points), each compared with no insomnia (0–7 points). The results suggest that higher physical activity levels are significantly linked to a lower risk of insomnia. This was especially true for women (OR = 0.59, 95% CI: 0.44–−0.81), people with primary school education or below (OR = 0.59, 95% CI: 0.42–−0.84), low-income groups earning less than 3,000 RMB per month (OR = 0.51, 95% CI: 0.34–−0.77), and nonsmokers (OR = 0.59, 95% CI: 0.44–−0.78). In men and those in higher-income or more educated groups, high physical activity also tended to reduce insomnia risk, but this difference was not statistically significant. On the other hand, sedentary behavior, especially sitting for more than 6 hours per day, was strongly linked to a greater risk of insomnia. This was particularly evident in women (OR = 2.01, 95% CI: 1.38–2.93), people with lower education levels (OR = 2.28, 95% CI: 1.43–3.62), low-income individuals (OR = 2.78, 95% CI: 1.66–4.68), and smokers (OR = 4.69, 95% CI: 1.09–5.44). These findings suggest that increasing physical activity and reducing sedentary time may be effective ways to lower the risk of insomnia, particularly in women, low-income, less-educated, and smoking populations (Table 4).

**Table 3:** Odds ratios for mild insomnia (8–14 vs. 0–7) and moderate-to-severe insomnia (15–28 vs. 0–7) by subgroup.

	*N* = 677	Physical activity		Sedentary behavior	
		Medium	High	3–6 h	≥6 h
Sex					
Female	531	**0.70 (0.51, 0,97)**	**0.59 (0.44, 0.81)**	**1.42 (1.09, 1.85)**	**2.01 (1.38, 2.93)**
Male	146	0.78 (0.47, 1.28)	0.65 (0.40, 1.08)	1.45 (0.87, 2.41)	1.77 (0.93, 3.37)
Education					
Primary school and below	360	0.70 (0.48, 1.01)	**0.59 (0.42, 0.84)**	**1.61 (1.15, 2.26)**	**2.28 (1.43, 3.62)**
Above primary school	317	0.75 (0.50, 1.11)	**0.63 (0.43, 0.94)**	1.21 (0.87, 1.68)	1.49 (0.95, 2.34)
Monthly family income					
<3,000 RMB	281	0.74 (0.49, 1.12)	**0.51 (0.34, 0.77)**	**1.55 (1.04, 2.30)**	**2.78 (1.66, 4.68)**
≥3,000 RMB	396	0.73 (0.51, 1.04)	**0.68 (0.48, 0.97)**	1.32 (0.99, 1.77)	1.40 (0.91, 2.14)
Current smoker					
Yes	86	0.90 (0.44, 1.84)	0.78 (0.38, 1.60)	**2.27 (1.09, 4.69)**	2.17 (0.86, 5.44)
No	591	**0.70 (0.52, 0.93)**	**0.59 (0.44, 0.78)**	**1.33 (1.03, 1.70)**	**1.93 (1.37, 2.74)**
Current drinker					
Yes	47	0.61 (0.25, 1.50)	0.53 (0.22, 1.29)	1.05 (0.48, 2.28)	1.03 (0.34, 3.13)
No	630	**0.75 (0.56, 0.99)**	**0.62 (0.47, 0.82)**	**1.46 (1.14, 1.88)**	**2.02 (1.44, 2.84)**

Reference group: no insomnia (0–7 points). Models for mild insomnia (8–14) and moderate-to-severe insomnia (15–28) are presented separately. The bold values indicate statistical significance (*p* < 0.05).

**Table 4:** Interaction effects between physical activity and sedentary behavior with different variables.

	B	WALD	*p*	OR	95% CI
Sex*Sedentary behavior	0.098	0.129	0.719	1.103	0.645–1.887
Sex*Physical activity	0.043	0.072	0.789	1.044	0.761–1.434
Education*Sedentary behavior	0.130	0.933	0.334	1.139	0.875–1.150
Education*Physical activity	−0.032	1.148	0.717	0.969	0.816–1.150
Monthly family income*Sedentary behavior	−0.110	1.148	0.284	0.895	0.732–1.096
Monthly family income*Physical activity	0.110	2.837	0.092	1.116	0.982–1.267
Current smoker*Sedentary behavior	−0.370	0.805	0.370	0.691	0.308–1.550
Current smoker*Physical activity	−0.128	0.256	0.613	0.880	0.537–1.442
Current drinker*Sedentary behavior	0.145	3.237	0.072	1.156	0.987–1.355
Current drinker*Physical activity	−0.005	0.000	0.989	0.995	0.527–1.879
Constant	0.692	1.359	0.244	1.999	

## 4 Discussion

From a public health perspective, these findings highlight the need for targeted interventions to promote physical activity and reduce sedentary behavior among middle-aged and older adults, particularly in high-risk subgroups such as women, low-income individuals, and those with lower educational attainment. Clinicians could integrate PA counseling into routine care, recommending at least 1,500 MET-min/week of moderate-to-vigorous activity to prevent insomnia. Public health programs might design community-based exercise initiatives (e.g., tai chi, brisk walking) and implement strategies to break up prolonged sitting (e.g., hourly movement reminders). Given the dose–response relationship observed, even modest reductions in sedentary time (e.g., <3 h/day) could yield meaningful sleep benefits. These interventions could not only improve sleep quality but also mitigate comorbidities such as depression and cardiovascular disease, aligning with broader healthy aging goals.

This study investigated how different levels of physical activity and sedentary behavior affect insomnia, revealing a strong link between these behaviors and insomnia risk in middle-aged and older adults. The results revealed that 18% of the population had insomnia symptoms, with 4.4% having moderate to severe insomnia. The participants in the insomnia group had much lower physical activity levels than did those without insomnia. The moderate to severe insomnia group averaged only 1737 MET-min per week, whereas the non-insomnia group averaged 3,066 MET-min. Longer sedentary time was also linked to higher insomnia risk, especially for those with moderate to severe insomnia, who spent an average of 3.9 h per day sitting, compared with 3.36 h for those without insomnia.

This study provides evidence supporting the protective role of PA against insomnia, which is consistent with previous research (35). Specifically, moderate PA has been linked to a 55% reduction in the risk of moderate to severe insomnia, reinforcing its established benefits on sleep quality. However, our findings indicated that higher levels of PA, approximately 3,066 MET-minutes per week, may be necessary to effectively reduce insomnia risk, especially among middle-aged and older adults with moderate to severe symptoms. This finding differed from earlier studies, which suggested that even light PA could improve sleep (36). The discrepancy may be due to the focus on middle-aged and older adults in this study, as they may require higher levels of PA to counter the effects of aging and chronic health conditions.

Concerning sedentary behavior, this study aligned with previous findings that prolonged sitting might negatively impact sleep (37, 38). Sitting for more than 3 hours per day has been found to increase the risk of moderate to severe insomnia (39). Sedentary behavior has also been linked to a decline in metabolism and increased inflammation (40), which may have stronger negative effects on middle-aged and older adults. Long periods of sitting could worsen insulin resistance and metabolic problems, which then harm sleep quality. Sedentary behavior might also worsen anxiety and depression, which exacerbates insomnia (41). Dogra et al. noted that too much sitting is tied to many health problems, especially in middle-aged and older adults, as it harms both physical health and mental well-being, leading to poorer sleep (42).

Our study also revealed that PA and SB might affect different socioeconomic groups in different ways. Higher levels of PA were especially helpful in protecting women, low-income individuals, and those with lower education from insomnia. These findings suggest that people from these groups might benefit the most from exercise programs. Research has shown that lower socioeconomic status is closely linked to higher rates of insomnia (43), which further highlights the need for targeted help for these high-risk groups (44, 45). Sedentary time had a stronger negative effect on women and smokers, likely due to specific physical or mental vulnerabilities in these groups. As a result, programs that focus on increasing physical activity and reducing sitting time could help reduce insomnia rates in these groups.

In our study, the link between exercise and mental health was further supported. Zhu et al. reported that physical activity improves cognitive function, suggesting that regular exercise could enhance brain function and reduce insomnia risk in middle-aged and older adults (46). Some studies have shown that exercise also helps improve mental health, which in turn improves sleep quality, in agreement with our findings (47, 48).

Research has suggested that increasing the variety and intensity of physical activity could help middle-aged and older adults improve their metabolism and lower insomnia risk (49). This finding is similar to our findings, highlighting the need for different types of exercise in future programs. Many studies have also shown that reducing sedentary behavior is linked to better sleep quality, stressing the importance of lifestyle changes for middle-aged and older adults (37).

Results of interaction effect analysis showed that no interaction terms involving gender, educational level, smoking/drinking status with PA or SB were statistically significant (all *p* > 0.05). This indicates these factors did not significantly alter the association patterns between PA/SB and insomnia—for example, high PA reduced insomnia risk and prolonged SB increased insomnia risk consistently in both men and women, with only subgroup differences in effect size.

This study had several limitations. First, this study adopted a cross-sectional design, which only identifies associations between PA, SB, and insomnia, but cannot confirm the direction of causality. For instance, it remains unclear whether low PA levels lead to insomnia, or if insomnia decreases PA engagement—bidirectional relationships may exist between these factors. This study did not adjust for potential confounders such as sleep timing (e.g., bedtime regularity, stability of the sleep–wake cycle), use of sleep-impacting medications (e.g., antihypertensives, antidepressants), or PA timing (e.g., evening vigorous PA vs. morning moderate PA). Previous studies have shown that evening vigorous PA (≥6 METs) may delay sleep onset, while regular bedtimes reduce insomnia risk. These factors may modify the associations between PA/SB and insomnia and should be controlled in future studies. To validate causal hypotheses, future studies should employ prospective cohort designs to track long-term changes in PA/SB levels and the incidence of insomnia over 1–3 years. Additionally, the study sample was restricted to middle-aged and older adults in Wuhan, China, excluding rural populations, residents from other provinces, and ethnic minority groups. As a megacity, Wuhan has distinct lifestyle characteristics that differ from rural areas; meanwhile, cultural practices of China’s ethnic minorities may modulate the associations between PA/SB and insomnia. These factors limit the generalizability of the results to middle-aged and older populations globally or to non-urban, non-Han Chinese populations. Finally, the study relied solely on self-reported data via the IPAQ-SF, without incorporating objective measures such as activity trackers or wearable devices. This limits the reliability of PA and SB assessments, as self-reports are susceptible to recall bias, social desirability bias, and inaccuracies in estimating activity duration and intensity. Additionally, the IPAQ-SF’s focus on “typical workdays” may not capture weekend behavioral patterns, and middle-aged and older adults may struggle to accurately recall weekly activity durations, especially during periods of fluctuating health. Future studies using objective monitoring tools could provide more precise data on movement patterns and strengthen the validity of such associations. In this study, the Mini-Mental State Examination was used as the cognitive screening tool to exclude individuals with apparent cognitive impairment. However, the MMSE has limited sensitivity for detecting mild cognitive impairment, and its performance is influenced by educational attainment, which may lead to misclassification. Moreover, the MMSE does not comprehensively assess all cognitive domains, particularly executive functioning and subtle attentional deficits. These limitations may have allowed a subset of participants with mild cognitive impairment to be classified as cognitively normal, potentially introducing residual confounding. Future research may benefit from incorporating more comprehensive cognitive assessments, such as the Montreal Cognitive Assessment, or combining subjective and objective cognitive measures to increase screening accuracy.

To address unresolved questions, future research should focus on three areas: (1) Longitudinal designs to verify causality: Track changes in PA/SB (e.g., transitioning from low to high PA, reducing SB from ≥6 to <3 h/day) and subsequent insomnia incidence over time, to confirm whether modifying PA/SB reduces insomnia risk. (2) Mechanisms underlying subgroup differences: For example, the stronger association between PA/SB and insomnia in women may relate to increased sensitivity of the sleep-regulatory center due to declining estrogen levels, while the association in low-income groups may stem from limited access to PA facilities or higher psychological stress—qualitative interviews combined with quantitative data are needed to explore these mechanisms. (3) Comparative studies of SB interventions: Evaluate whether “breaking prolonged SB” (e.g., 5-min light activity breaks hourly) has a greater impact on insomnia than “reducing total SB duration,” to identify optimal SB modification strategies.

## 5 Conclusion

In conclusion, high levels of PA were associated with lower odds of mild and moderate-to-severe insomnia, whereas moderate PA was associated with lower odds of moderate-to-severe insomnia but not mild insomnia. Sedentary behavior of ≥3 h/day was associated with increased odds of insomnia, with the highest risk observed at ≥6 h/day. Further prospective studies in a larger population are warranted to explore causes and effects and further clarify the differences between groups.

## Data Availability

The original contributions presented in the study are included in the article/supplementary material, further inquiries can be directed to the corresponding authors.
